# NMR Characterization of Conformational Interconversions of Lys48-Linked Ubiquitin Chains

**DOI:** 10.3390/ijms21155351

**Published:** 2020-07-28

**Authors:** Methanee Hiranyakorn, Saeko Yanaka, Tadashi Satoh, Thunchanok Wilasri, Benchawan Jityuti, Maho Yagi-Utsumi, Koichi Kato

**Affiliations:** 1 Department of Functional Molecular Science, School of Physical Science, The Graduate University for Advanced Studies (SOKENDAI), 5-1 Higashiyama, Myodaiji, Okazaki, Aichi 444-8787, Japan; methanee@ims.ac.jp (M.H.); saeko-yanaka@ims.ac.jp (S.Y.); 2 Institute for Molecular Science (IMS), National Institutes of Natural Sciences, 5-1 Higashiyama, Myodaiji, Okazaki, Aichi 444-8787, Japan; meawzabaii@hotmail.com (T.W.); bengy_cassi@hotmail.com (B.J.); 3 Exploratory Research Center on Life and Living Systems (ExCELLS), National Institutes of Natural Sciences, 5-1 Higashiyama, Myodaiji, Okazaki, Aichi 444-8787, Japan; 4 Graduate School of Pharmaceutical Sciences, Nagoya City University, 3-1 Tanabe-dori, Mizuho-ku, Nagoya 467-8603, Japan; tadashisatoh@phar.nagoya-cu.ac.jp

**Keywords:** Lys48-linked ubiquitin chains, NMR, multidomain protein, cyclic protein

## Abstract

Ubiquitin (Ub) molecules can be enzymatically connected through a specific isopeptide linkage, thereby mediating various cellular processes by binding to Ub-interacting proteins through their hydrophobic surfaces. The Lys48-linked Ub chains, which serve as tags for proteasomal degradation, undergo conformational interconversions between open and closed states, in which the hydrophobic surfaces are exposed and shielded, respectively. Here, we provide a quantitative view of such dynamic processes of Lys48-linked triUb and tetraUb in solution. The native and cyclic forms of Ub chains are prepared with isotope labeling by in vitro enzymatic reactions. Our comparative NMR analyses using monomeric Ub and cyclic diUb as reference molecules enabled the quantification of populations of the open and closed states for each Ub unit of the native Ub chains. The data indicate that the most distal Ub unit in the Ub chains is the most apt to expose its hydrophobic surface, suggesting its preferential involvement in interactions with the Ub-recognizing proteins. We also demonstrate that a mutational modification of the distal end of the Ub chain can remotely affect the solvent exposure of the hydrophobic surfaces of the other Ub units, suggesting that Ub chains could be unique design frameworks for the creation of allosterically controllable multidomain proteins.

## 1. Introduction

Sophisticated protein functions are, in many cases, mediated through the cooperative interplay between two or more domains [1]. Proteins with a modular architecture of multiple domains connected by linkers often exhibit diversity in relative positions of the individual domains organized through weak and even transient inter-domain interactions [1,2]. Therefore, in order to extend our understanding of the working mechanisms of multidomain proteins in living systems, the quantitative characterization of their conformational interchanges in solution is necessary.

**Figure 1:**
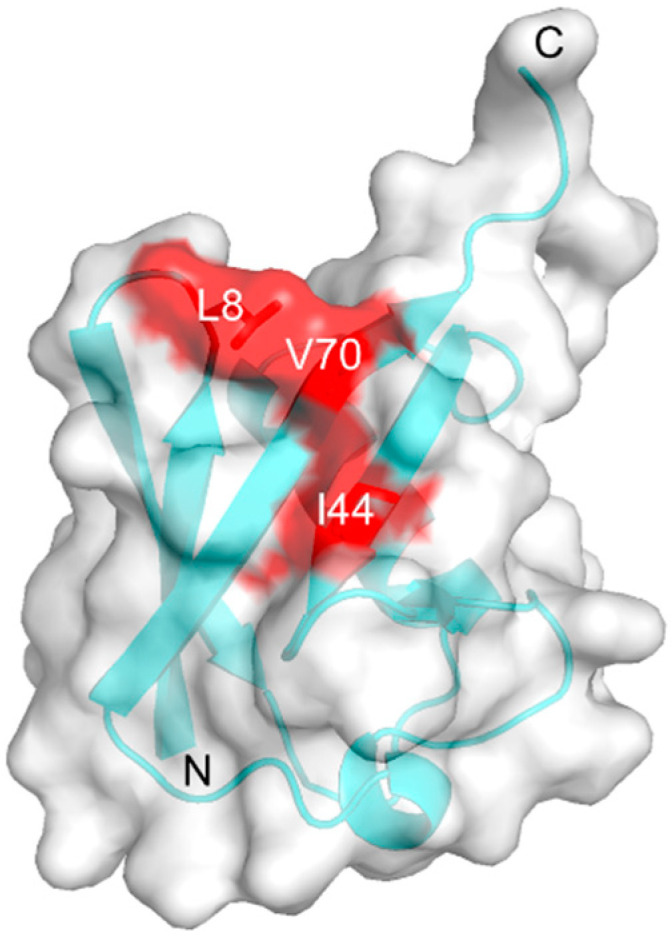
Crystal structure of ubiquitin (Ub; PDB: 1UBQ) [26] highlighting Leu8, Ile44, and Val70 on the hydrophobic surface with a transparent surface model presentation.

Polymeric ubiquitin (Ub) chains, in which several Ub proteins are connected through specific isopeptide bonds, are known to play regulatory roles in various cellular processes, including cell cycle progression, DNA repair, transcriptional regulation, and apoptosis [3,4,5]. The Ub chains conjugated by different linkages carry distinct biological information in the form of a “Ub code” that is read out by specific Ub-interacting proteins [3,6]. These proteins generally recognize the hydrophobic surfaces displayed on the Ub chains. The Lys48-linked Ub chain serves as a tag for protein degradation by the 26S proteasome [7], whereas the Lys63-linked Ub chain is involved in nonproteolytic functions, such as signaling in DNA repair and transcriptional regulation [8,9]. The proteins that recognize the specific Ub chains interact with a hydrophobic surface, which includes Leu8, Ile44, and Val70 of Lys48-linked Ub [6,10] (Figure 1). The Lys63-linked diUb and tetraUb chains exhibit extended conformations in which the hydrophobic surfaces are exposed to a solvent, as revealed by crystallographic and NMR studies [11,12,13]. In contrast, the crystal structures of Lys48-linked Ub chains often exhibit closed conformations, in which the hydrophobic patches are shielded due to Ub-Ub interactions in the chains [14,15]. In addition, their dynamic domain rearrangements have been observed in solution using NMR spectroscopy [16,17,18,19,20,21], single-molecule FRET [22] and molecular simulation [23,24,25]. For example, our previous NMR study enabled the quantitative characterization of the conformational interchange of the native form of Lys48-linked diUb (n-diUb) between the open and closed conformations, based on conventional chemical shift data [20].

Here, we extend our previous work by characterizing conformational interconversions of the native forms of Lys48-linked triUb (n-triUb) and tetraUb (n-tetraUb) chains in a solution. Using enzymatically synthesized Ub chains in cyclic and native (non-cyclic) forms, conformational equilibria of Ub chains in terms of the exposure of the hydrophobic surfaces are quantitatively delineated based on the NMR spectroscopic data.

## 2. Results and Discussion

### 2.1. Spectral Comparison Among Cyclic Forms of Lys48-Linked diUb, triUb, and tetraUb

**Figure 2:**
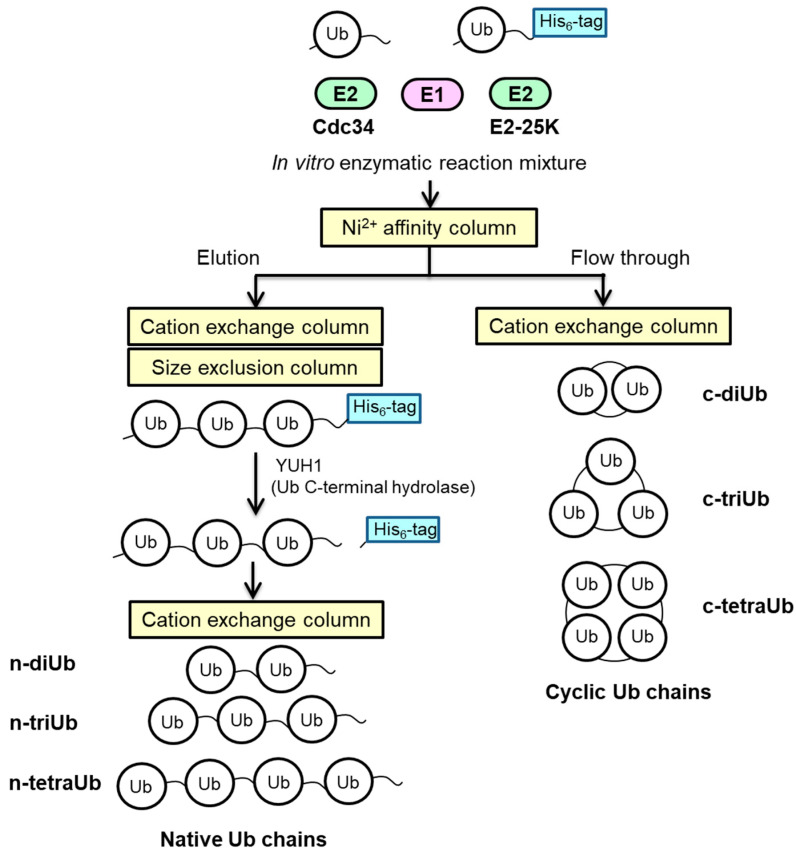
Schematic diagram of the preparation of native and cyclic forms of Lys48-linked Ub chains.

We prepared the n-triUb and n-tetraUb in native and cyclic forms by in vitro enzymatic reactions and chromatographic separation (Figure 2). In the cyclic forms, the C-terminus of one Ub unit is conjugated to the Lys48 of its most distal Ub unit.

**Figure 3:**
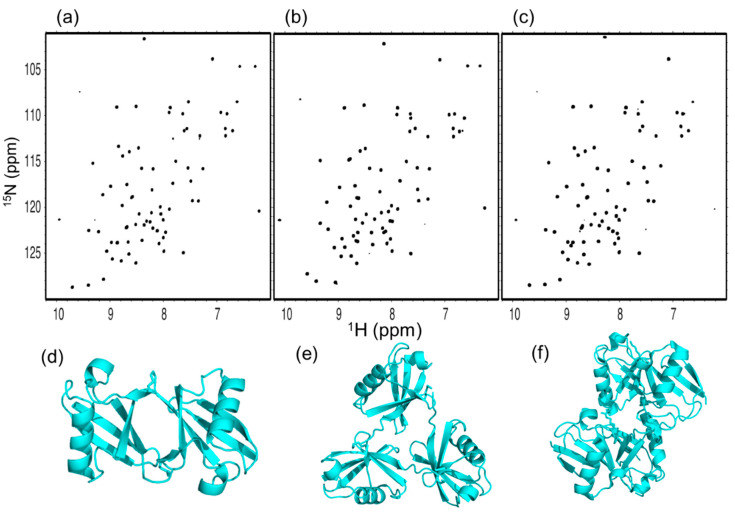
^1^H-^15^N HSQC spectra of uniformly ^15^N-labeled (**a**) c-diUb, (**b**) c-triUb, and (**c**) c-tetraUb. 3D structural models of (**d**) c-diUb [20], (**e**) c-triUb (solved in this study, PDB: 7CAP), and (**f**) c-tetraUb (PDB: 3ALB) [27].

We first compared the ^1^H-^15^N HSQC spectra of the cyclic forms of Lys48-linked diUb (c-diUb), triUb (c-triUb), and tetraUb (c-tetraUb) (Figure 3a–c and Figure S1a). Each of these cyclic forms gave a single set of chemical shifts, indicating their symmetrical structures. The ^1^H-^15^N HSQC spectra of the c-tetraUb and c-diUb were well-superimposed, indicating their similarity with respect to the Ub–Ub interaction (Figure S1b). This is consistent with our previously reported structures of c-diUb and c-tetraUb, which share almost identical closed conformations [20,27] (Figure 3d,f and Figure S2). In contrast, the spectrum of c-triUb was significantly different from that of c-diUb in terms of the chemical shifts of the peaks originating from the hydrophobic surface and the segment linking the Ub units (Figure S1a,c). We determined a crystal structure of c-triUb at a resolution of 1.33 Å (Figure 3e and Figure S3 and Table S1). As expected from the NMR spectra, c-triUb adopts a three-fold symmetric structure in which the hydrophobic surfaces, including Leu8, Ile44, and Val70, are partially exposed, distinct from the closed conformation observed for c-diUb and c-tetraUb.

### 2.2. Spectral Comparison among Native Forms of Lys48-Linked diUb, triUb, and tetraUb

**Figure 4:**
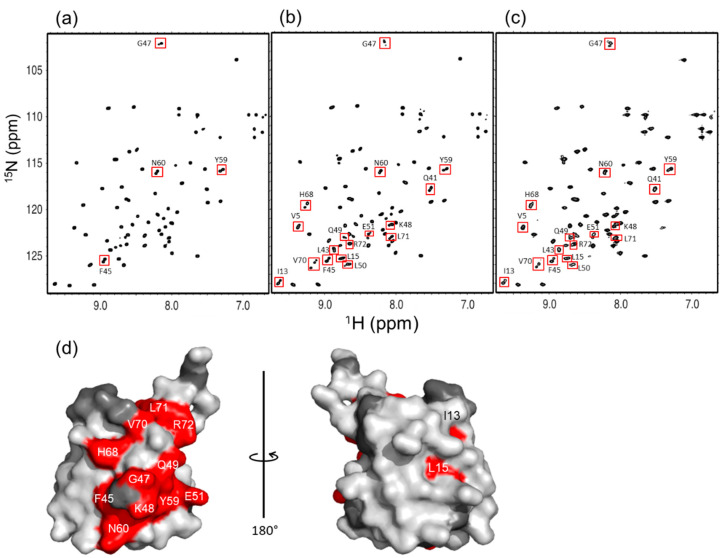
^1^H-^15^N HSQC spectra of uniformly ^15^N-labeled (**a**) n-diUb, (**b**) n-triUb, and (**c**) n-tetraUb. Multiple peaks (boxed) are displayed. (**d**) Mapping of the 3D structure of monomeric Ub (PDB: 1UBQ), with residues showing the multiple peaks. The proline residues and the residues whose ^1^H-^15^N HSQC peaks could not be observed as probes because of broadening and/or overlapping are shown in gray.

In the following, we compare the ^1^H-^15^N HSQC spectra of n-diUb, n-triUb, and n-tetraUb (Figure 4a–c). As we reported previously [20], n-diUb has a single set of chemical shifts, except for the peaks originating from the Lys48-Gly76 linkage site, indicating that the two Ub units in n-diUb are structurally equivalent. In contrast, n-triUb and n-tetraUb exhibited multiple peaks for many residues, suggesting differences in the local environment among the Ub units. For the spectral assignments, we prepared a series of n-triUb and n-tetraUb analogs, in which a specific Ub unit was isotopically labeled. Each of these proteins exhibited a single set of chemical shifts in its ^1^H-^15^N HSQC spectrum (Figure S4). The amino acid residues showing different chemical shifts among different Ub units were located in the hydrophobic surfaces, as exemplified by Val70, in addition to those located in the Lys48-Gly76 linkage site (Figure 4d). These observations suggest that the hydrophobic surface of the Ub units in the n-triUb and n-tetraUb experience different environments in time average.

### 2.3. Conformational Equilibrium of the Native Forms of Lys48-Linked triUb and tetraUb

**Figure 5:**
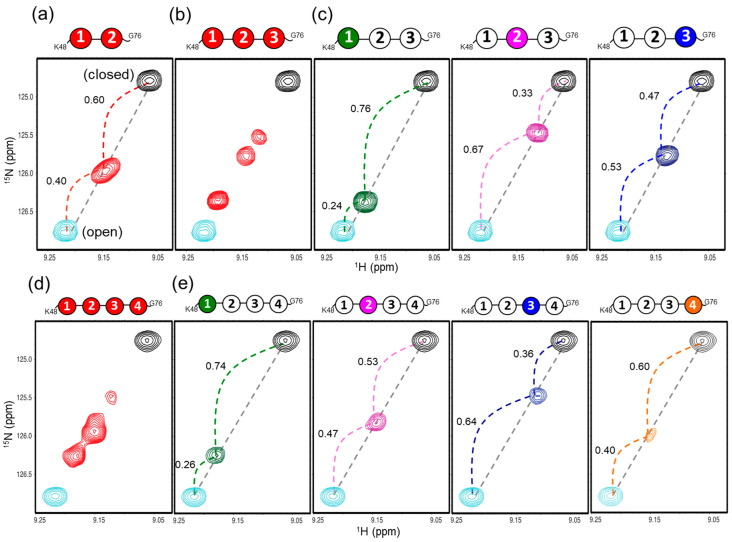
^1^H-^15^N HSQC peaks originating from Val70 of (**a**) uniformly ^15^N-labeled n-diUb (red), (**b**) uniformly^15^N-labeled n-triUb (red), (**c**) unit-selectively ^15^N-labeled n-triUb chains at the distal Ub1 (green), the middle Ub2 (magenta) and the proximal Ub3 (blue), (**d**) uniformly ^15^N-labeled n-tetraUb (red), and (**e**) unit-selectively ^15^N-labeled n-tetraUb at the distal Ub1 (green), the middle Ub2 (magenta), the middle Ub3 (blue), and the proximal Ub4 (orange). The peaks from the monomeric Ub (open form) and c-diUb (closed form) are plotted in cyan and black, respectively. The dividing ratios of the chemical shift differences of n-diUb, n-triUb, and n-tetraUb are indicated between monomeric Ub and c-diUb.

Our previous NMR study revealed that n-diUb undergoes a conformational transition in the fast exchange regime between the open and closed states, which is mimicked by monomeric Ub and c-diUb, respectively, in terms of the exposure of the hydrophobic surfaces to the solvent [20]. Namely, c-diUb exhibits a closed conformation, in which two hydrophobic surfaces are highly packed against each other, whereas the hydrophobic surface of the monomeric Ub is exposed to the solvent. Under this circumstance, each amino acid residue located on the hydrophobic surfaces of the Ub units gave an HSQC peak between the peaks originating from the corresponding residues in monomeric Ub and c-diUb in the same straight line, as exemplified by the Val70 of n-diUb (Figure 5a). This provided an opportunity to estimate the population of each conformation based on the internal division ratios by the n-diUb’s peak of the chemical shift difference between monomeric Ub and the c-diUb, with the assumption that they represent fully open and closed states, respectively.

Intriguingly, this approach was applicable for estimating the conformer populations of n-triUb and n-tetraUb, because the multiple peaks originating from the hydrophobic surfaces of the Ub units in these Ub chains were also aligned in the same straight line between the corresponding peaks from c-diUb and monomeric Ub, exhibiting varying degrees of line broadening (Figure 5b–e). It should be noted that c-triUb was not suitable as a model of the closed state, because of its exposed hydrophobic surfaces (Figure 3e), which resulted in a significant chemical shift deviation from the straight line, as exemplified by the Val70 peak (Figure S5). These NMR data indicate that each Ub unit of n-triUb and n-tetraUb experienced a dynamic transition in the moderately fast exchange between the open and closed states on the relevant NMR timescale (approximately 100 Hz). Under this condition, the population of each conformer can be estimated on the basis of dividing the ratios of the chemical shift differences, using the monomeric Ub and c-diUb as references.

**Figure 6:**
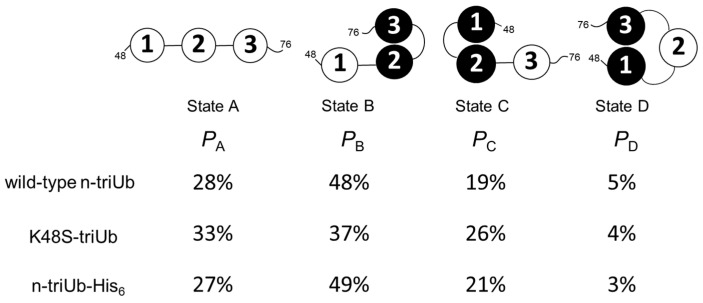
Cartoon model of the conformational equilibrium of n-triUb. The populations of states A, B, C, and D of n-triUb are denoted as *P*_A_, *P*_B_, *P*_C_, and *P*_D_, respectively. The calculated *P*_A_, *P*_B_, *P*_C_, and *P*_D_ values of wild-type n-triUb, K48S-triUb, and n-triUb-His_6_ are indicated.

In n-triUb, the dividing ratio between the monomeric and cyclic forms in the Val70 peak indicated that the populations of open and closed conformers made up 76% and 24% of the total conformers in the distal unit (Ub1), 33% and 67% in the middle unit (Ub2), and 47% and 53% in the proximal unit (Ub3), respectively (Figure 5c and Figure S6). These data indicate that the hydrophobic surface of the distal unit (Ub1) is mostly exposed to the solvent, while that of the middle unit (Ub2) is mostly shielded. To consider the overall open–closed conformational equilibrium state of n-triUb, we formed a hypothetical model with four different conformational states (Figure 6), and estimated their populations based on the three different dividing ratios of the chemical shift differences of the Val70 peak, according to the following equations:

Ub1 open:closed = *P*_A_ + *P*_B_:*P*_C_ + *P*_D_ = 0.76:0.24 (1)

Ub2 open:closed = *P*_A_ + *P*_D_:*P*_B_ + *P*_C_ = 0.33:0.67 (2)

Ub3 open:closed = *P*_A_ + *P*_C_:*P*_B_ + *P*_D_ = 0.47:0.53 (3)

*P*_A_ + *P*_B_ + *P*_C_ + *P*_D_ = 1 (4)

where *P*x is the population of state x.

According to the calculation results, the population of state A (fully open state) was 28% and those of state B, state C, and state D (three different closed states) were 48%, 19%, and 5%, respectively (Figure 6). This indicates that the “end-to-end” Ub interaction leaving the middle Ub2 unit open (state D) seldom occurs, presumably due to steric restriction, rendering Ub2 mostly involved in the interaction with either Ub1 or Ub3. It should be noted that the population of state B is significantly higher than that of state C, suggesting that Ub2 has a higher affinity for Ub3 than for Ub1.

In a similar way, we estimated the populations of the open and closed conformers of n-tetraUb based on dividing ratios with respect to the Val70 peak. The populations of the open state of Ub1, Ub2, Ub3, and Ub4 were estimated as 74%, 53%, 36%, and 60%, respectively (Figure 5e and Figure S7). Namely, the distal Ub1 unit has a more highly solvent-exposed hydrophobic surface than the remaining Ub units, as in the case of n-triUb.

### 2.4. Inter-Subunit Interactions of Lys48-Linked triUb

To explain the higher open-state propensity of the distal Ub units in n-triUb and n-tetraUb, we considered the possible end effects attributed to the distal and proximal end of the Ub chain, i.e., the Lys48 amino group of the distal Ub and the C-terminal carboxyl group of proximal Ub. The hydrophobic patch of Ub is surrounded by basic amino acid residues, i.e., Lys6, Arg42, Lys48, His68, and Arg72, which are likely to destabilize the closed conformation due to electrostatic repulsion. Indeed, our previous study demonstrated that the substitution of His68 with valine resulted in a significant increase in the population of the closed state in n-diUb [20]. The previously reported simulation data also showed that electrostatic interactions made a negative contribution to the formation of compact Lys48-linked diUb [23].

**Figure 7:**
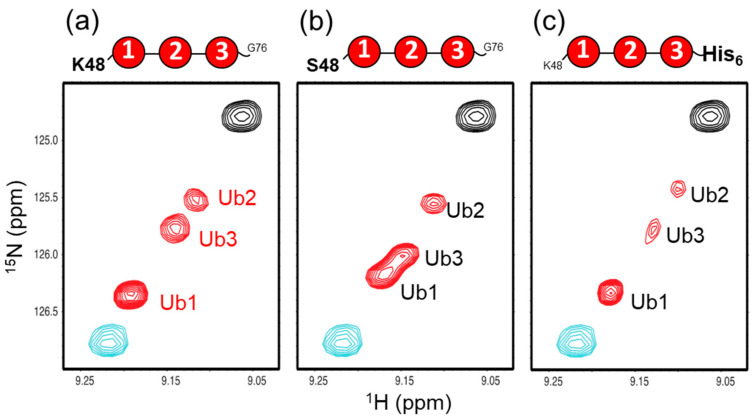
^1^H-^15^N HSQC peaks originating from Val70 of uniformly ^15^N-labeled (**a**) wild-type n-triUb, (**b**) K48S-triUb, and (**c**) n-triUb-His_6_. The peaks from monomeric Ub and c-diUb are shown in cyan and black, respectively.

Here, we examined the possible effect of the substitution of Lys48 with serine in the distal Ub of n-triUb (K48S-triUb) on its conformational states. Fortunately, the chemical shifts of the monomeric Ub were hardly affected by this mutation, except with respect to the proximity of the mutation site (Figure S8a,c), allowing us to use wild-type c-diUb and wild-type monomeric Ub as the reference molecules for mimicking the closed and open states. The multiple peaks from the Val70 of the K48S-triUb exhibited clearly different chemical shifts, but were still aligned in the same straight line (Figure 7b). The chemical shift of each Ub unit of the K48S-triUb was assigned using the different combinations of the unit-selective ^15^N-labeled analogs, thereby enabling the estimation of the population of each conformational state (Figure S9). The calculated populations of states A, B, C, and D were 33%, 37%, 26%, and 4%, respectively (Figure 6). As compared to the wild-type n-triUb, the population of state C was increased by the mutation (from 19% to 26%), with a concomitant decrease of population B (from 48% to 37%), indicating that the Lys48 of the distal Ub negatively contributes to its closed state formation.

We also attempted to examine a possible C-terminal effect by using an n-triUb analog with a C-terminal extension with hexahistidine in the proximal Ub (n-triUb-His_6_), which was also subjected to the NMR-based conformer population quantification. The multiple peaks from the Val70 of n-triUb-His_6_ were distributed in the same straight line (Figure 7c and Figure S8b,d). According to the calculated population, no significant difference was observed in each of the conformer populations between n-triUb and n-triUb-His_6_ (Figure 6). These data demonstrate that the positively charged Lys48 in the distal Ub affected the conformer population distribution of n-triUb, while the proximal C-terminal group had little impact on it.

## 3. Materials and Methods

### 3.1. Expression and Purification of Human Ub and Derivatives

Human Ub and C-terminally hexahistidine-tagged Ub (Ub-His_6_) were expressed and purified as described previously [20]. The K48S Ub mutant was generated using site-directed mutagenesis techniques, and was purified by the same protocol used for the wild-type Ub. Briefly, the wild-type and mutated Ub were expressed from pGEX6p-1 plasmid in *Escherichia coli* BL21(DE3) CodonPlus cells, which were grown in M9 minimal media containing [^15^N]NH_4_Cl (1 g/L) in order to produce the isotopically labeled protein. After sonication, the supernatant was purified using a DEAE column in a buffer of 50 mM Tris-HCl (pH 8.0). The pH of the collected flow-through was adjusted to 4.5 by using 10% acetic acid. After centrifugation, the supernatant was purified in a 20 mM sodium acetate (pH 4.5) using a SP-TOYOPEARL column (TOSOH, Japan). Finally, the buffer was exchanged with 50 mM Tris-HCl (pH 8.0).

### 3.2. Expression and Purification of Ub-Related Enzymes

Ub E1, E2-25K, and yeast ubiquitin hydrolase 1 (YUH1) were expressed and purified as described previously [20]. The plasmid vector encoding the Cdc34 E2 ubiquitin-conjugating enzyme was constructed and cloned as a fusion protein with glutathione S-transferase-tagged Cdc34 (GST-Cdc34) using the pCold vector. Subsequently, the vector was transformed into *E. coli* strain BL21-CodonPlus (Agilent Technologies, Santa Clara, CA, USA). The transformed bacteria were grown at 37 °C in LB medium containing 50 μg/mL ampicillin, until the OD = 0.65 at 600 nm, and then the cultures were rapidly cooled in an ice bath for 10–15 min. The GST-Cdc34 protein was induced with 0.5 mM IPTG and the cultures were incubated at 15 °C overnight. The cell pellets were dissolved in 20 mM Tris-HCl (pH 8.0), 150 mM NaCl, and were disrupted by sonication. GST-Cdc34 was purified using a glutathione affinity column (GE Healthcare, Chicago, IL, USA). The Cdc34 protein was enzymatically cleaved from GST-Cdc34 by incubation with TEV protease. The cleaved Cdc34 was purified using a glutathione column, again, in order to remove the GST-tag, and was then concentrated up to 2 mg/mL in the presence of 0.5 mM dithiothreitol (DTT), and stored at −80 °C.

### 3.3. Enzymatic Synthesis of Lys48-Linked tri-Ub

The cyclic and native forms of the Lys48-linked Ub chains were prepared by an in vitro enzymatic reaction using E2-25K and Cdc34, respectively (Figure 2). The cyclic Lys48-linked Ub chains were prepared using E2-25K, as described previously [20]. We used Cdc34, because it enables the selective production of native Lys48-linked Ub chains [28]. [^15^N]Ub-His_6_ and [^15^N]Ub were mixed at a molar ratio of 2:1 in 50 mM Tris-HCl (pH 8.0) and incubated at 37 °C for 16 h in the presence of 0.6 µM E1, 20 µM Cdc34, 1 mM DTT, 5 mM MgCl_2_, 10 mM ATP, 0.6 units/mL creatine phosphokinase, and 10 mM creatine phosphate. After the reaction, the Lys48-linked Ub chains possessing the hexahistidine tag were separated using Mono S (GE Healthcare) cation exchange chromatography. For cleaving the hexahistidine tag, the Ub chains were treated with YUH1. The Ub chains without the hexahistidine tag were purified by MonoS cation exchange chromatography and size exclusion chromatography (Superdex 75; GE Healthcare). The protein purity was verified by SDS-PAGE. For n-triUb and n-tetraUb chains, subunit-specifically ^15^N-labeled chains were prepared through an in vitro enzymatic reaction using a ^15^N-labeled or unlabeled Ub/Ub-His_6_. The subunit at the distal end of the Ub chain was termed Ub1 and the remaining subunits were numbered sequentially.

### 3.4. NMR Measurements

All of the NMR samples were prepared in a 10 mM sodium phosphate buffer at pH 7.0 with 95% H_2_O/5% D_2_O (*v*/*v*). The NMR spectra were recorded at 42 °C using Bruker AVANCE-III HD 500 and AVANCE 800US spectrometers equipped with a 5-mm cryogenic triple-resonance probe. The data were processed using NMR Topspin (Bruker, Billerica, MA, USA) and analyzed using Sparky [29].

### 3.5. Crystallization, X-Ray Data Collection, and Structure Determination

For the crystallization, purified c-triUb was dissolved in a protein concentration of 8.0 mg/mL in 20 mM Tris-HCl (pH 7.5) and 150 mM NaCl. Protein crystals were obtained in a buffer containing 20% PEG3350 and 200 mM zinc acetate with incubation at 20 °C, and cryoprotected with a crystallization buffer supplemented with 20% glycerol. The diffraction data were integrated and scaled using HKL2000 [30]. The crystals of c-triUb belonged to space group *C*2 and were diffracted up to a resolution of 1.33 Å. The crystal structure of c-triUb was solved by the molecular replacement method using the program MOLREP [31] with a human ubiquitin (Protein Data Bank code 1UBQ) as a search model. Model fitting to the electron density maps and the subsequent refinement were conducted using COOT [32] and REFMAC5 [33], respectively. The stereochemical quality of the final model was validated using MolProbity [34]. The data collection and refinement statistics of c-triUb are summarized in Table S1. The molecular graphics were prepared using PyMOL (Schrödinger, New York, NY, USA).

### 3.6. Accession Number

The coordinates and structural factors of the crystal structure of the cyclic Lys48-linked triUb were deposited in the Protein Data Bank under accession number 7CAP.

## 4. Concluding Remarks

Our NMR results provide a quantitative view of the conformational multiplicities of Lys48-linked triUb and tetraUb chains, which undergo dynamic domain rearrangement between the open and closed states in a solution. While the hydrophobic surfaces recognized by the Ub-interacting proteins are shielded in the closed states [10], the domain rearrangement coupled with their exposure is likely an indispensable step for opening the Ub codes, as well as the enzymatic elongation and processing of Ub chains [35,36,37]. The present data show that the most distal Ub unit in the Lys48-linked Ub chains is the most apt to expose its hydrophobic surface to a solvent, suggesting its preferential involvement in molecular recognition processes. The present data also demonstrate that the mutational modification of the distal end of the n-triUb chain remotely affects the solvent exposure of the hydrophobic surfaces of the other Ub units. This allostery depends on the competitive sharing of Ub2 between Ub1 and Ub3 in the formation of closed states. In longer Ub chains, Ub3 could be shared, at least, between Ub2 and Ub4. Thus, the mutational effect at the most distal Ub (Ub1) is allosterically transmitted to the remaining Ub units in a chain-reaction manner. This suggests that the Lys48-linked Ub chains may offer unique design frameworks for creating allosterically controllable multidomain proteins.

## Supplementary Materials

Supplementary materials can be found at https://www.mdpi.com/1422-0067/21/15/5351/s1. Figure S1. Spectral comparison of the cyclic Ub chains. (a) Superposition of the ^1^H-^15^N HSQC spectra of c-diUb (black), c-triUb (red), and c-tetraUb (cyan). Chemical shift differences (b) between c-tetraUb and c-diUb, and (c) between c-triUb and c-diUb. Data are shown according to the equation (0.04*δ*_N_^2^ + *δ*_H_^2^)^1/2^, where *δ*_N_ and *δ*_H_ represent the difference in nitrogen and proton chemical shifts, respectively. The proline residues and the residues whose ^1^H-^15^N HSQC peak could not be used as a probe because of broadening are shown by asterisks; Figure S2. Structural similarity among (a) the closed conformation of n-diUb observed in crystals [PDB: 1AAR] [1], (b) the NMR structure of c-diUb [2], and (c) an n-diUb part derived from the c-tetraUb crystal structure [PDB: 3ALB] [3]. The 3D structure models were shown with the same orientations, highlighting the positions of Val70 in red. Lys48 and Gly76 forming the isopeptide bond are shown as stick models; Figure S3. Crystal structure of c-triUb (solved in this study; PDB: 7CAP) highlighting Leu8, Ile44, and Val70 on the hydrophobic surface; Figure S4. ^1^H-^15^N HSQC spectra of n-triUb chains, which were unit-selectively ^15^N-labeled at (a) the distal Ub1 (green), (b) the middle Ub2 (magenta), and (c) the proximal Ub3 (blue). ^1^H-^15^N HSQC spectra of n-tetraUb chains, which were unit-selectively ^15^N-labeled at (d) the distal Ub1 (green), (e) the second Ub2 (magenta), (f) the third Ub3 (blue), and (g) the proximal Ub4 (orange); Figure S5. ^1^H-^15^N HSQC peaks originating from Val70 of monomeric Ub (cyan), c-diUb (black), c-triUb (red), and unit-selectively ^15^N-labeled n-triUb chains at the distal Ub1 (green), the middle Ub2 (magenta) and the proximal Ub3 (blue); Figure S6. Conformer populations n-triUb estimated from the NMR spectral data. A cartoon model of the possible conformers in each state is shown. A pair of Ub units whose hydrophobic surfaces are shielded from each other are shown in black; Figure S7. Conformer populations of n-tetraUb estimated from the NMR spectral data. A cartoon model of the possible conformers in each state is shown. A pair of Ub units whose hydrophobic surfaces are shielded from each other are shown in black; Figure S8. ^1^H-^15^N HSQC spectra of monomeric Ub (blue) with (a) monomeric K48S-Ub (red) and (b) monomeric Ub-His_6_ (red). Chemical shift differences (c) between monomeric Ub and monomeric K48S-Ub, and (c) between monomeric Ub and monomeric Ub- His_6_. Data are shown according to the equation (0.04*δ*_N_^2^ + *δ*_H_^2^)^1/2^, where *δ*_N_ and *δ*_H_ represent the difference in nitrogen and proton chemical shifts, respectively. The proline residues and the residues whose ^1^H-^15^N HSQC peak could not be used as a probe because of broadening are shown by asterisks; Figure S9. ^1^H-^15^N HSQC peaks originating from Val70 of (a) uniformly ^15^N-labeled K48S-triUb, (b) unit-selectively ^15^N-labeled K48S-triUb chains at Ub2 and Ub3, and (c) unit-selectively ^15^N-labeled K48S-triUb at Ub1 and Ub2; Table S1. Data collection and refinements statistics for the crystal structure of c-triUb.

## Abbreviations

DTT	Dithiothreitol
GST	Glutathione S-transferase
HSQC	Heteronuclear Single Quantum Correlation
NMR	Nuclear magnetic resonance
Ub	Ubiquitin
YUH	Yeast ubiquitin hydrolase
