# Supplementary material for: NMR Characterization of Conformational Interconversions of Lys48-Linked Ubiquitin Chains
Source: Int J Mol Sci. 2020 Jul 28;21(15):5351. doi: 10.3390/ijms21155351 (PMC7432494; doi:10.3390/ijms21155351)
Supplement: Supplementary file 1 [file ijms-21-05351-s001.pdf]

Supplementary Material

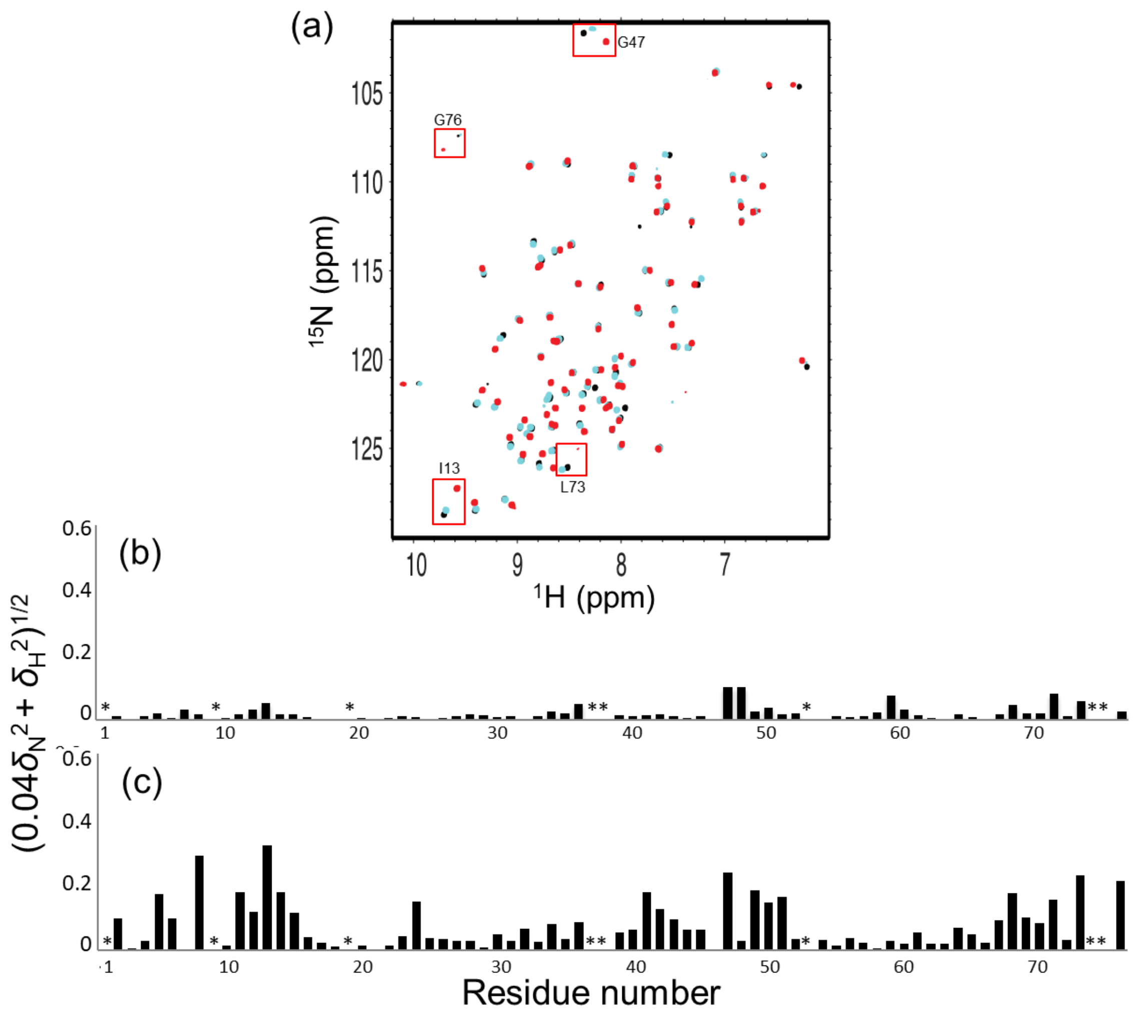

Figure S1. Spectral comparison of the cyclic Ub chains. (a) Superposition of the ^{1}H-^{15}N HSQC spectra of c-diUb (black), c-triUb (red), and c-tetraUb (cyan). Chemical shift differences (b) between c-tetraUb and c-diUb, and (c) between c-triUb and c-diUb. Data are shown according to the equation $(0.04\delta_N^2 + \delta_H^2)^{1/2}$, where δ_N and δ_H represent the difference in nitrogen and proton chemical shifts, respectively. The proline residues and the residues whose ^{1}H-^{15}N HSQC peak could not be used as a probe because of broadening are shown by asterisks.

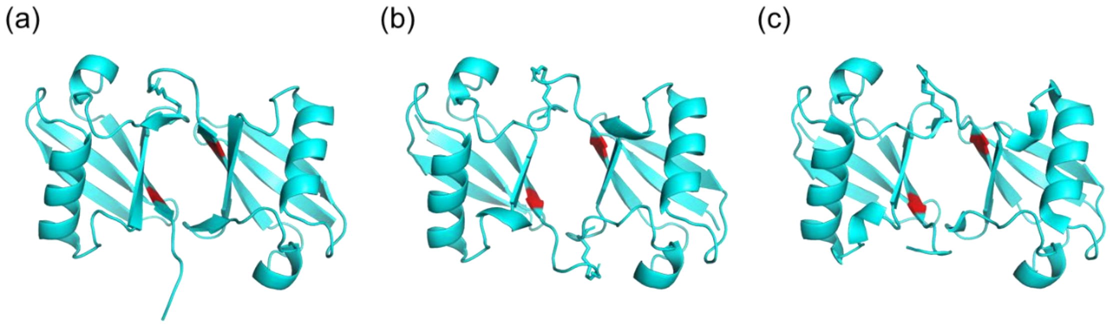

Figure S2. Structural similarity among (a) the closed conformation of n-diUb observed in crystals [PDB: 1AAR] [1], (b) the NMR structure of c-diUb [2], and (c) an n-diUb part derived from the c-tetraUb crystal structure [PDB: 3ALB] [3]. The 3D structure models were shown with the same orientations, highlighting the positions of Val70 in red. Lys48 and Gly76 forming the isopeptide bond are shown as stick models.

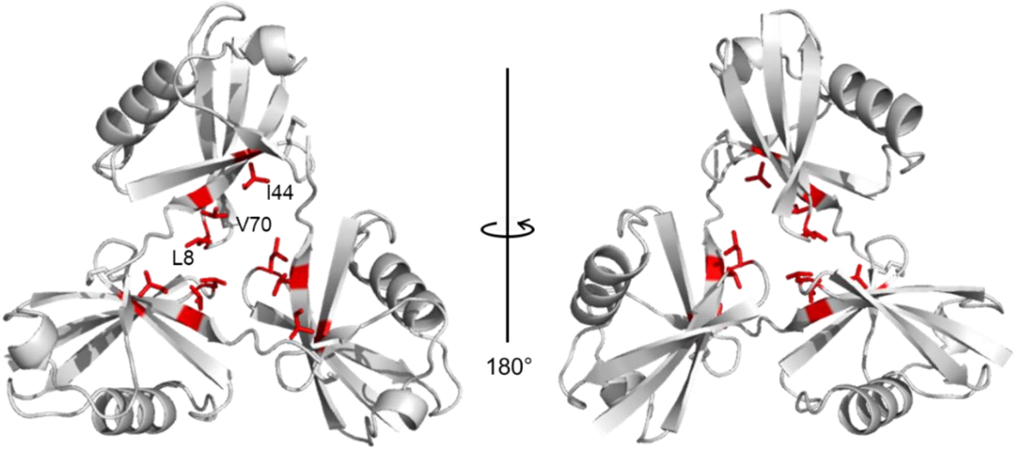

Figure S3. Crystal structure of c-triUb (solved in this study; PDB: 7CAP) highlighting Leu8, Ile44, and Val70 on the hydrophobic surface.

Table S1. Data collection and refinements statistics for the crystal structure of c-triUb.

**Crystallographic data**	
Space group	*C*2
Unit cell *a*/*b*/*c* (Å)	88.4/54.8/61.0
β (˚)	122.0
**Data processing statistics**	
Beam line	Photon Factory AR-NE3A
Wavelength (Å)	1.0000
Resolution (Å)	50–1.33 (1.35–1.33)
Total/unique reflections	285,708/56,837
Completeness (%)	99.9 (100.0)
R_{merge} (%)	7.5 (78.4)
$I/\sigma(I)$	25.2 (1.5)
**Refinement statistics**	
Resolution (Å)	20.0–1.33
R_{work}/R_{free} (%)	13.5/17.4
RMS deviations from ideal	
Bond lengths (Å)	0.014
Bond angles (˚)	1.74
Ramachandran plot (%)	
Favored	100
Allowed	0
Outliers	0

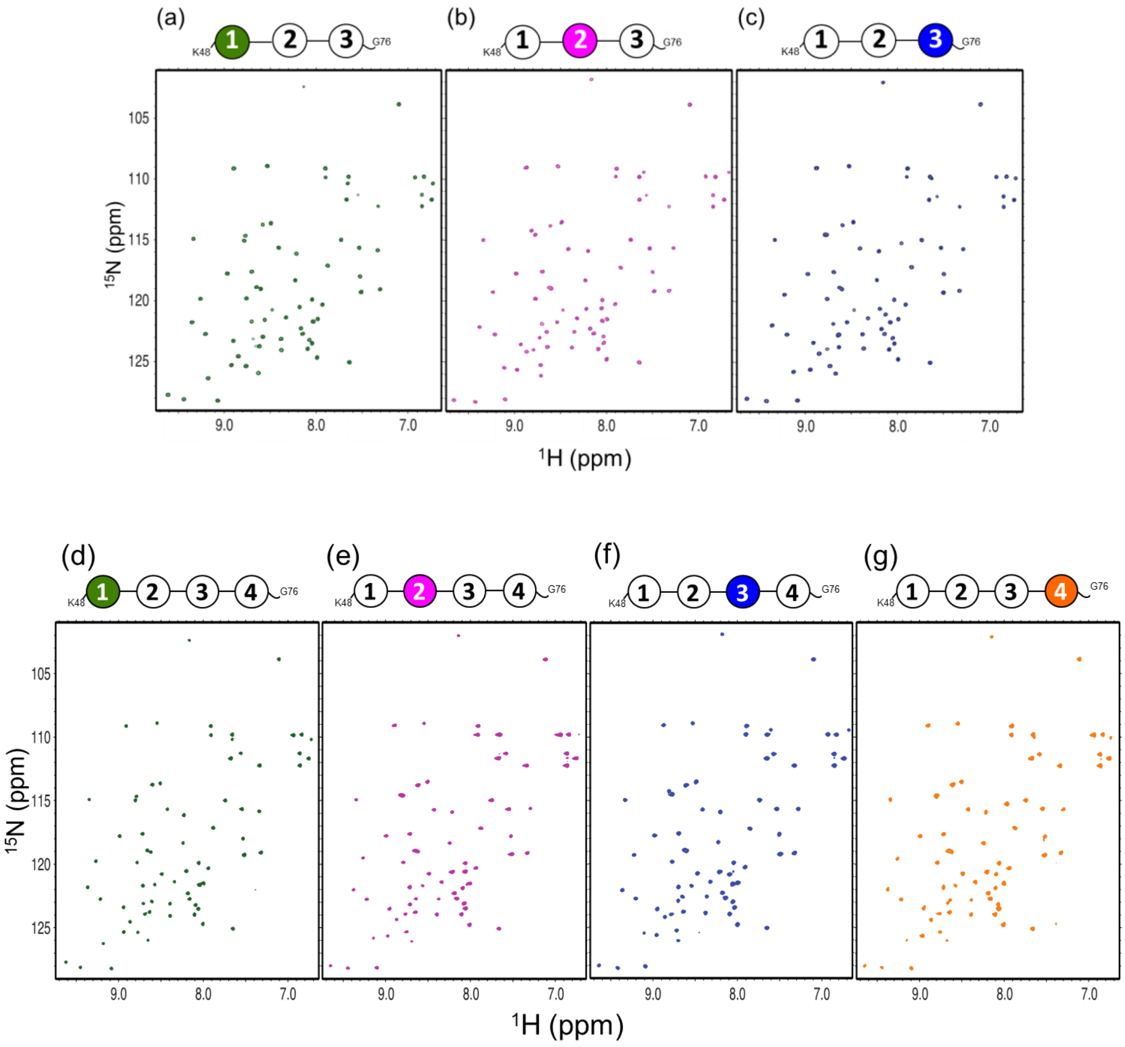

Figure S4. ^{1}H-^{15}N HSQC spectra of n-triUb chains, which were unit-selectively ^{15}N-labeled at (a) the distal Ub1 (green), (b) the middle Ub2 (magenta), and (c) the proximal Ub3 (blue). ^{1}H-^{15}N HSQC spectra of n-tetraUb chains, which were unit-selectively ^{15}N-labeled at (d) the distal Ub1 (green), (e) the second Ub2 (magenta), (f) the third Ub3 (blue), and (g) the proximal Ub4 (orange).

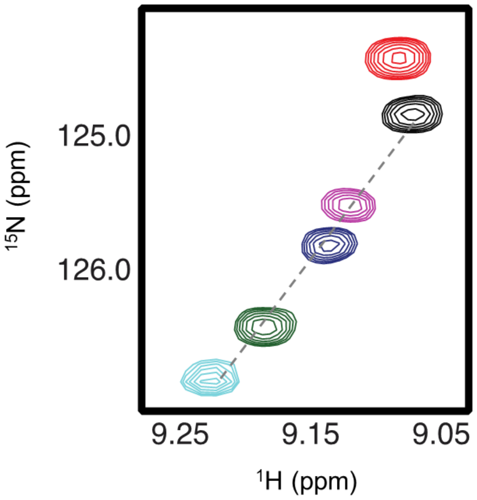

Figure S5. ^{1}H-^{15}N HSQC peaks originating from Val70 of monomeric Ub (cyan), c-diUb (black), c-triUb (red), and unit-selectively ^{15}N-labeled n-triUb chains at the distal Ub1 (green), the middle Ub2 (magenta) and the proximal Ub3 (blue).

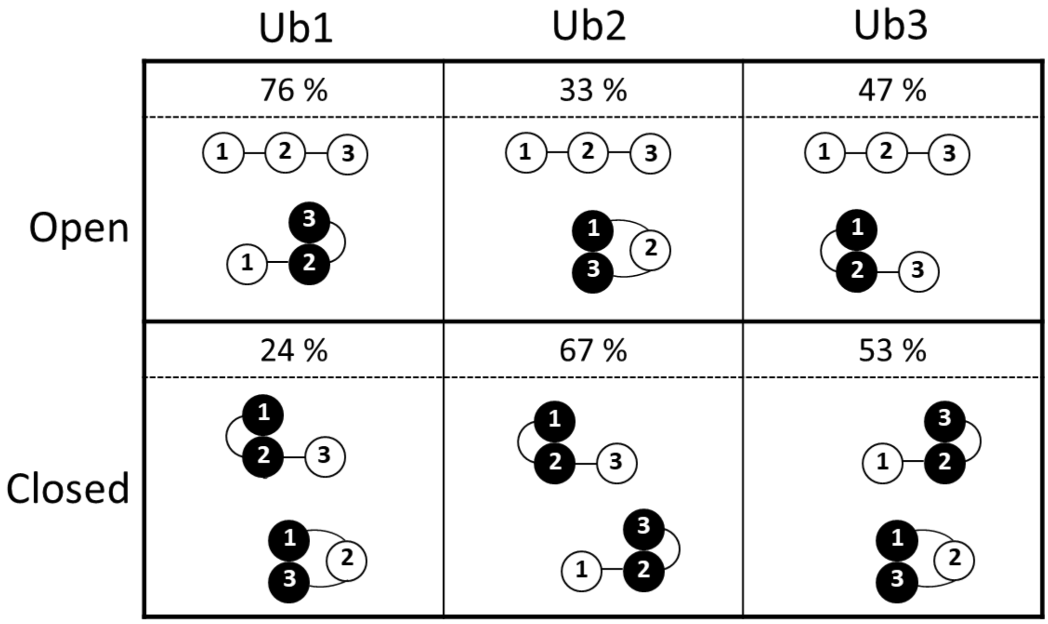

Figure S6. Conformer populations n-triUb estimated from the NMR spectral data. A cartoon model of the possible conformers in each state is shown. A pair of Ub units whose hydrophobic surfaces are shielded from each other are shown in black.

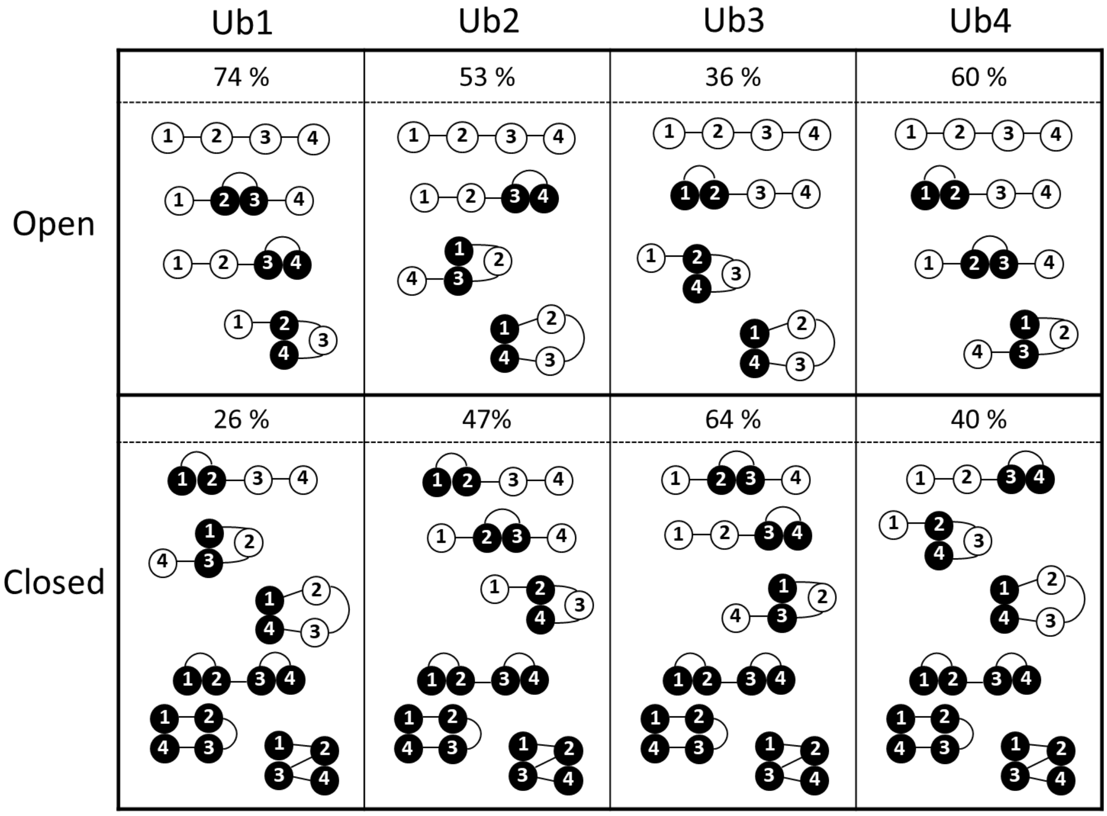

Figure S7. Conformer populations of n-tetraUb estimated from the NMR spectral data. A cartoon model of the possible conformers in each state is shown. A pair of Ub units whose hydrophobic surfaces are shielded from each other are shown in black.

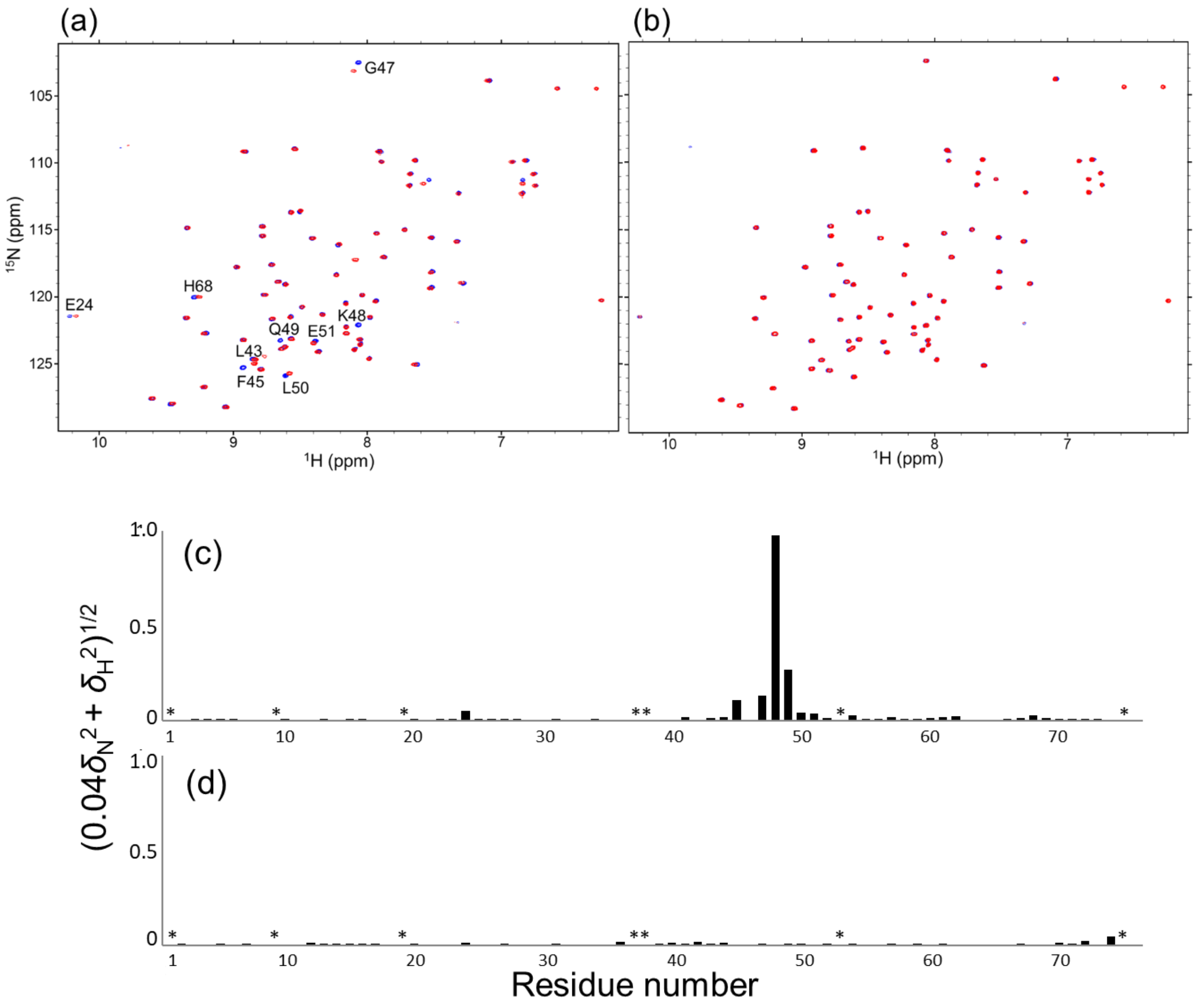

Figure S8. ^{1}H-^{15}N HSQC spectra of monomeric Ub (blue) with (a) monomeric K48S-Ub (red) and (b) monomeric Ub-His$_6$ (red). Chemical shift differences (c) between monomeric Ub and monomeric K48S-Ub, and (c) between monomeric Ub and monomeric Ub- His$_6$. Data are shown according to the equation $(0.04\delta_N{}^2 + \delta_H{}^2)^{1/2}$, where δ_N and δ_H represent the difference in nitrogen and proton chemical shifts, respectively. The proline residues and the residues whose ^{1}H-^{15}N HSQC peak could not be used as a probe because of broadening are shown by asterisks.

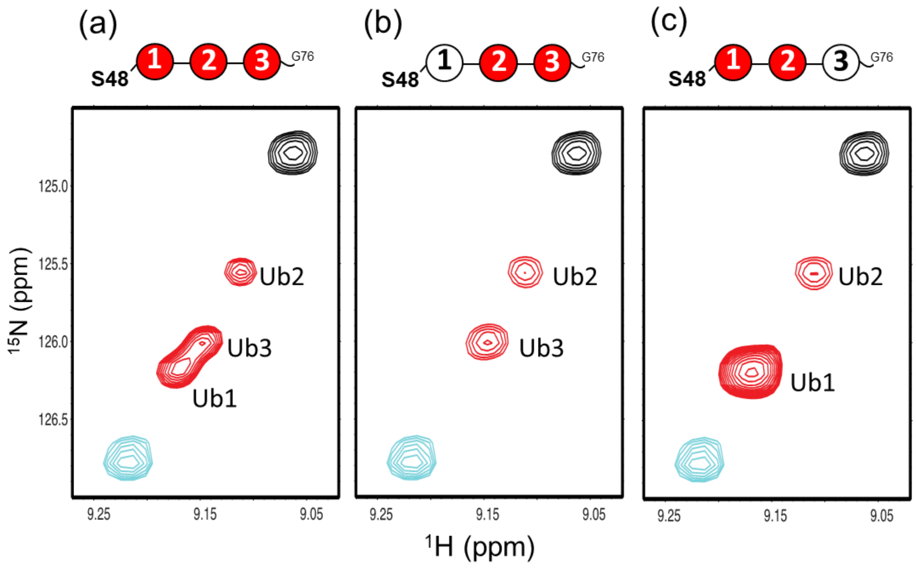

Figure S9. ^{1}H-^{15}N HSQC peaks originating from Val70 of (a) uniformly ^{15}N-labeled K48S-triUb, (b) unit-selectively ^{15}N-labeled K48S-triUb chains at Ub2 and Ub3, and (c) unit-selectively ^{15}N-labeled K48S-triUb at Ub1 and Ub2.

References

1. Cook, W. J.; Jeffrey, L. C.; Carson, M.; Chen, Z.; Pickart, C. M., Structure of a diubiquitin conjugate and a model for interaction with ubiquitin conjugating enzyme (E2). *J Biol Chem* **1992,** 267, (23), 16467-71.
2. Hirano, T.; Serve, O.; Yagi-Utsumi, M.; Takemoto, E.; Hiromoto, T.; Satoh, T.; Mizushima, T.; Kato, K., Conformational dynamics of wild-type Lys-48-linked diubiquitin in solution. *J Biol Chem* **2011,** 286, (43), 37496-502.
3. Satoh, T.; Sakata, E.; Yamamoto, S.; Yamaguchi, Y.; Sumiyoshi, A.; Wakatsuki, S.; Kato, K., Crystal structure of cyclic Lys48-linked tetraubiquitin. *Biochem Biophys Res Commun* **2010,** 400, (3), 329-33.
